# Glutamatergic Neurons in the Cerebellar Lateral Nucleus Contribute to Motor Deficits Induced by Chronic Sleep Disturbance

**DOI:** 10.3390/brainsci15111185

**Published:** 2025-10-31

**Authors:** Jian Zhu, Wan-Qiao Qi, Ling-Xi Kong, Yan-Mei Lin, Feng-Fei Ding, Zhi-Li Huang, Wei-Min Qu

**Affiliations:** 1 Department of Pharmacology, School of Basic Medical Sciences, Fudan University, Shanghai 200032, China; 21111010115@m.fudan.edu.cn (J.Z.); 21111010111@m.fudan.edu.cn (L.-X.K.); fengfei_ding@fudan.edu.cn (F.-F.D.); huangzl@fudan.edu.cn (Z.-L.H.); 2 Department of Neurology, The Second Affiliated Hospital of Harbin Medical University, Harbin 150018, China; qiwanqiao1997@163.com; 3 Department of Psychiatry, Sleep Medicine Center, Nanfang Hospital, Southern Medical University, Guangzhou 510515, China; lymsmu@163.com; 4 State Key Laboratory of Medical Neurobiology and MOE Frontiers Centre for Brain Science, Institutes of Brain Science, Joint International Research Laboratory of Sleep, Fudan University, Shanghai 200032, China; 5 Department of Anaesthesiology, Zhongshan Hospital, Fudan University, Shanghai 200032, China

**Keywords:** cerebellum, deep cerebellar nuclei, lateral nucleus, glutamatergic neurons, chronic sleep disruption, motor dysfunction

## Abstract

**Background/Objectives**: The cerebellum is essential for motor coordination and has recently been implicated in sleep-related disorders. However, the neural mechanisms linking sleep disruption to motor dysfunction remain poorly understood. This study aimed to elucidate the roles of the deep cerebellar nuclei (DCN), particularly the lateral nucleus, in motor dysfunction induced by chronic sleep disruption (CSD). **Methods**: Using a validated mouse model of CSD with periodic sleep fragmentation induced by an orbital shaker during the light phase, we assessed neuronal activation via c-Fos immunostaining and performed chemogenetic manipulation of glutamatergic neurons within the lateral nucleus. Behavioral performance was evaluated using open-field and rotarod tests. **Results**: CSD selectively increased c-Fos expression in the lateral nucleus, with no significant changes observed in other DCN subregions. Chemogenetic activation or ablation of glutamatergic neurons in the lateral nucleus decreased locomotor activity in the open-field test and shortened latency to fall in the rotarod task. Conversely, chemogenetic inhibition of these neurons attenuated CSD-induced impairments, restoring locomotor performance toward control levels. **Conclusions**: Our findings provide direct experimental evidence that glutamatergic neurons in the lateral nucleus play a crucial role in mediating CSD-induced motor dysfunction. These results highlight the cerebellar contribution to the interplay between sleep and motor control and identify a potential target for therapeutic intervention in sleep-related motor disorders.

## 1. Introduction

Sleep is a highly conserved physiological state that is essential for neural plasticity, cognition, metabolism, and immune regulation [1,2,3]. Disruption of sleep architecture, such as sleep fragmentation or poor sleep quality, is closely associated with dysfunctions in neural, metabolic, and immune systems [4,5]. While sleep facilitates the consolidation of motor skills [6], the impact of chronic sleep disruption (CSD) on motor performance has been relatively underexplored. Clinical studies have shown that patients with obstructive sleep apnea (OSA) exhibit deficits in motor sequence memory [7], and experimental studies in rodents, including our previous work, have demonstrated that CSD impairs motor coordination and exploratory behavior [8]. However, the neural mechanisms underlying the link between sleep disruption and motor dysfunction remain poorly understood.

The cerebellum plays a pivotal role in motor control and coordination [9]. Beyond refining voluntary movements, both structural and functional cerebellar abnormalities have been associated with various sleep disorders [10], suggesting that the cerebellum may serve as an integrative hub connecting sleep regulation with motor output. The deep cerebellar nuclei (DCN), comprising lateral, interposed, and medial nuclei [11,12,13], constitute the primary output structures of the cerebellum. Among these, the lateral nucleus (dentate nucleus in primates) is the largest and most evolutionarily advanced, playing a critical role in motor planning, initiation, and fine-tuning [14,15]. The lateral nucleus contains three principal neuronal subtypes—glutamatergic, GABAergic, and glycinergic—with glutamatergic projection neurons providing the predominant excitatory output to thalamic and brainstem motor centers [13,16]. Their activity is tightly regulated by inhibitory inputs from Purkinje cells, and disruption of this balance can result in motor impairments such as ataxia, tremor, or reduced movement precision [17,18,19].

Based on these considerations, we hypothesized that motor deficits induced by CSD are mediated through altered activity of glutamatergic neurons in the lateral nucleus. To test this hypothesis, we examined whether CSD modifies neuronal activity in this region and whether direct manipulation of glutamatergic projection neurons is sufficient to modulate motor performance. We found that CSD elicited robust c-Fos expression selectively within the lateral nucleus. Chemogenetic activation of glutamatergic neurons in the lateral nucleus was sufficient to reproduce key motor impairments, including impaired coordination and decreased locomotor activity. Conversely, chemogenetic inhibition of these neurons alleviated CSD-induced impairments, restoring locomotor performance toward control levels. To our knowledge, this study provides the first direct evidence that glutamatergic neurons in the cerebellar lateral nucleus play a crucial role in mediating motor dysfunction resulting from chronic sleep disruption. These findings offer novel insights into the mechanisms of sleep–motor coupling and identify a potential therapeutic target for sleep-related motor disorders.

## 2. Materials and Methods

### 2.1. Animals

Adult male C57BL/6J mice (8–12 weeks old) were obtained from Shanghai Jie Si Jie Laboratory Animal Co., Ltd. (Shanghai, China) Vglut2-IRES-Cre mice (Slc17a6^tm2(cre)Lowl^, The Jackson Laboratory, Farmington, CT, USA, Stock No. 017535) [20] were provided by Dr. Hu Ji (Shanghai Tech University, Shanghai, China). Male heterozygous Vglut2-IRES-Cre mice (10–24 weeks old, C57BL/6J background) were used for all experiments. Animals were housed under standard laboratory conditions: a 12-h light/dark cycle (lights on at 07:00, defined as zeitgeber time 0, ZT0; lights off at 19:00, ZT12), with an ambient temperature of 22 ± 1 °C, and relative humidity of 50–60%. Food and water were available ad libitum unless otherwise specified. All experimental procedures were conducted in accordance with the guidelines of the Medical Experimental Animal Administrative Committee of Shanghai. Protocols were approved by the Animal Ethics Committee of the School of Basic Medical Sciences, Fudan University (approval number: 20200306-023). Every effort was made to minimize animal suffering and to use the minimum number of animals required to obtain reliable and reproducible results.

### 2.2. Chronic Sleep Disruption Model

Mice underwent electroencephalogram (EEG) and electromyogram (EMG) electrode implantation followed by a recovery period of at least seven days. They were then randomly assigned to one of two groups: a control group with undisturbed sleep and a group exposed to chronic sleep disruption for seven consecutive days (CSD 7d). The CSD paradigm followed a previously validated method [8,21], in which mice remained in their home cages placed on an orbital platform shaker. The shaker was programmed to rotate at 110 rpm in cycles of 20 s on and 100 s off, operating daily during the light phase (ZT0–ZT12). From ZT12–ZT24, the shaker was inactive to allow normal sleep. Animals had unrestricted access to food and water throughout the procedure.

For chemogenetic inhibition experiments during CSD, mice were allowed at least three weeks for viral expression before being randomly assigned to three groups: (1) mCherry-Ctrl (control, no CSD), (2) mCherry-CSD (control virus + CSD), and (3) hM4Di-CSD (hM4Di + CSD). Both CSD groups underwent the 7-day CSD paradigm described above. During this period, animals received daily intraperitoneal injections of clozapine-N-oxide (CNO, C4759, LKT Laboratory, Saint Paul, MN, USA, 1 mg/kg) at 9:00 (ZT2). Behavioral testing was conducted after completion of the 7-day CSD protocol.

### 2.3. EEG and EMG Electrode Implantation

For EEG and EMG recordings, mice were surgically implanted with electrodes under deep anesthesia. Anesthesia was induced with 3% and maintained with 1.5% isoflurane in oxygen-enriched air. Mice were secured in a stereotaxic frame (RWD, Shenzhen, China), and body temperature was maintained at 37 ± 0.5 °C with a thermostatically controlled heating pad throughout the procedure. As previously described [22,23], two stainless steel screw electrodes were implanted over the right hemisphere using a cranial drill for EEG recordings. The frontal electrode was positioned 1.0 mm anterior to Bregma and 1.5 mm lateral to the midline, while the parietal electrode was placed 1.0 mm anterior to Lambda and 1.5 mm lateral to the midline. For EMG recordings, two insulated stainless-steel wires were inserted into the dorsal neck muscles to monitor muscle tone. All electrodes were secured to the skull with dental cement.

### 2.4. Polysomnographic Recordings and Analysis

Throughout the 7-day CSD procedure, sleep disturbance was induced using an orbital platform shaker operating exclusively during the light phase (ZT0–ZT12). On days 5 and 6, mice were connected to the recording cable and acclimated to the recording chamber during the dark phase (ZT12–ZT24) to ensure proper adaptation to the recording environment. After completing the CSD protocol on day 7, mice were immediately connected to the polysomnographic recording system at ZT12. EEG and EMG signals were recorded using a lightweight and flexible cable connected to a slip ring to allow unrestricted movement. The signals were amplified and filtered (EEG: 0.5–25 Hz; EMG: 20–200 Hz) using a Biotex amplifier (Kyoto, Japan). Signals were then digitized at a sampling rate of 128 Hz and recorded with Vital Recorder software (version 1.8.2.506, Kissei Comtec, Nagano, Japan). Polysomnographic recordings were performed continuously for at least 24 h, covering both light and dark phases. Sleep–wake states were manually scored offline in 4-s epochs using SleepSign software (version 3.0, Kissei Comtec, Nagano, Japan) according to standard criteria [24,25]. Each epoch was classified as wakefulness, rapid eye movement (REM) sleep, and non-rapid eye movement (NREM) sleep. Wakefulness was identified by low-amplitude, high-frequency EEG with concurrent high EMG tone; NREM sleep was characterized by high-amplitude, low-frequency (delta band: 0.5–4 Hz) EEG with reduced EMG tone; REM sleep was defined by regular theta activity (6–10 Hz) and muscle atonia.

### 2.5. Surgeries and Viral Injections

Stereotaxic surgeries were performed under isoflurane anesthesia (3% for induction, 1.5% for maintenance) in oxygen-enriched air. Mice were placed in a stereotaxic frame (RWD, Shenzhen, China), and body temperature was maintained at 37 ± 0.5 °C using a feedback-controlled heating pad. Prior to incision, the scalp was sterilized with alternating swabs of 75% ethanol and povidone–iodine. A small craniotomy was drilled above the target region, and viral vectors were delivered using a pulled glass micropipette connected to a microsyringe pump (Nanoject III, Drummond, Birmingham, AL, USA). For chemogenetic manipulation, mice received bilateral injections of either rAAV-EF1α-DIO-hM3Dq-mCherry (for neuronal activation, titer ~ 5.27 × 10^12^ vg/mL), rAAV-EF1α-DIO-hM4Di-mCherry (for neuronal inhibition, titer ~ 5.18 × 10^12^ vg/mL), or rAAV-EF1α-DIO-mCherry (control, titer ~ 5.13 × 10^12^ vg/mL). For ablation experiments, the experimental group received a 2:1 mixture of rAAV-EF1α-flex-taCasp3-TEVp (titer ~ 5.12 × 10^12^ vg/mL) and rAAV-EF1α-DIO-mCherry, while the control group received the control virus alone. All AAV vectors were obtained from BrainVTA (Shumi Technology, Wuhan, China) and were bilaterally injected into the lateral nucleus of Vglut2-IRES-Cre mice. The stereotaxic coordinates relative to Bregma were as follows: anteroposterior (AP), –6.0 mm; mediolateral (ML), ±2.45 mm; dorsoventral (DV), –3.5 mm. A total volume of 70 nL was infused at a rate of 2 nL/s, and the micropipette was left in place for 10 min after injection to minimize backflow. Following surgery, the scalp was sutured and mice were returned to a heated recovery cage. Postoperative care included subcutaneous analgesia with meloxicam (5 mg/kg) and daily monitoring for signs of distress. Mice were allowed to recover for at least 3 weeks to ensure sufficient viral expression before behavioral or physiological experiments.

### 2.6. Behavioral Assessment

#### 2.6.1. Open-Field Test

The open-field test (OFT) was performed to evaluate spontaneous locomotor activity and anxiety-like behavior. Mice were individually placed in the center of a square open field arena (50 × 50 × 40 cm, opaque white acrylic) under uniform illumination (~50 lux). Each session lasted 30 min, during which animals were allowed to explore freely. To assess anxiety-like behavior, the first 5 min of the session was analyzed, focusing on the time spent in central zone and the number of entries into the zone. A reduction in either parameter was interpreted as an indicator of heightened anxiety-like behavior. In addition, the full 30-min session was analyzed to evaluate locomotor activity, quantified by the total distance traveled and the percentage of active time. Animal movement was recorded using an overhead video camera and analyzed with an automated tracking system (Tracking Master V3.0). The arena was virtually divided into peripheral and central zones, with the central zone defined as the central 30 × 30 cm area. To eliminate olfactory interference from residual odor cues, the arena was wiped with 75% ethanol and dried between each trial. All behavioral tests were performed in an isolated, sound-attenuated room with constant lighting conditions during the light phase (ZT6–ZT10). To minimize external disturbance, the experimenter remained outside the room throughout the testing period. For chemogenetic activation, mice received an intraperitoneal injection of CNO (1 mg/kg) 30 min prior to testing. Experimenters were blinded to group assignments throughout both testing and data analysis.

#### 2.6.2. Rotarod Test

The rotarod test was performed to assess motor coordination, balance and motor learning. Mice were tested on a five-lane accelerating rotarod apparatus (SANS Bio-Tech, Nantong, Jiangsu, China), as previously described [26] with minor modifications. Prior to testing, all animals were habituated to the apparatus with two training trials conducted at a constant speed of 4 rpm, each lasting a maximum of 120 s, with a 15-min inter-trial interval. For the testing phase, the rod accelerated linearly from 4 to 40 rpm over a 5-min period. Each mouse underwent three trials per day for three consecutive days, with at least 15 min between trials. The latency to fall was recorded for each trial and used for analysis. All tests were conducted during the light phase (ZT6–ZT10) under consistent environmental conditions. For chemogenetic activation experiments, mice received an intraperitoneal injection of CNO (1 mg/kg) 30 min before testing. The experimenters were blinded to the group assignments throughout the procedures and data analysis.

#### 2.6.3. Elevated Plus Maze Test

The elevated plus maze (EPM) test was performed to assess anxiety-like behavior in mice. The apparatus consisted of two open arms (30 × 5 cm) and two closed arms of the same size with 15-cm high opaque walls, arranged in a plus-shaped configuration and elevated 50 cm above the floor. The maze was positioned in a quiet, dimly lit room (~50 lux). At the start of each trial, mice were placed in the center of the maze facing an open arm and allowed to explore freely for 5 min. Behavioral activity was recorded using an overhead video camera and analyzed with automated tracking software (Tracking Master V3.0). The time spent in open and closed arms was used as an index of anxiety-like behavior, with reduced time or fewer entries into the open arms interpreted as indicators of increased anxiety. The number of entries into each arm type was quantified to assess exploratory activity, with an entry defined as all four paws entering an arm. To minimize olfactory interference from residual odor cues, the apparatus was cleaned with 75% ethanol between trials. All tests were conducted during the light phase (ZT6–ZT10). Experimenters were blinded to group allocation throughout testing and data analysis.

#### 2.6.4. Y Maze Test

The Y maze test was performed to assess spatial working memory and spontaneous alternation behavior. The apparatus consisted of three identical arms (each 35 cm long and 8 cm wide, with 15 cm high walls) arranged at 120° angles to form a Y-shaped configuration. The test was conducted in a quiet, dimly lit room (~50 lux) to minimize stress-induced behavioral variability. At the start of each session, mice were placed at the end of one arm and allowed to explore the maze freely for 8 min. Animal movements were recorded using an overhead video camera and analyzed offline with automated tracking software (Tracking Master V3.0). Spontaneous alternation was defined as consecutive entries into all three distinct arms (e.g., A–B–C or C–B–A) without revisiting the same arm, regardless of turning direction. The percentage of spontaneous alternations was calculated using the formula: [Number of alternations/(Total arm entries–2)] × 100. Higher alternation percentages are considered indicative of better spatial working memory. The maze was thoroughly cleaned with 75% ethanol between trials to eliminate olfactory cues. All tests were performed during the light phase (ZT6–ZT10), and experimenters were blinded to group assignments throughout all procedures and data analyses.

### 2.7. Immunofluorescence

For immunostaining of c-Fos, mCherry, and NeuN, mice were deeply anesthetized with sodium pentobarbital (60 mg/kg, i.p.) and transcardially perfused with 40 mL phosphate-buffered saline (PBS), followed by 40 mL of 4% paraformaldehyde (PFA) in PBS. Brains were carefully removed, post-fixed overnight in 4% PFA at 4 °C, and cryoprotected in 30% sucrose in phosphate buffer until they sank. Coronal brain sections (30 μm thick) were prepared using a freezing microtome (CM1950, Leica, Wetzlar, Germany) and collected in four series. Free-floating sections were washed in PBS and incubated in blocking solution containing 0.3% Triton X-100 and 5% donkey serum in PBS for 1 h at room temperature. Primary antibodies diluted in the same blocking buffer were applied and incubated overnight at 4 °C. After primary incubation, sections were washed three times (10 min each) in PBS containing 0.3% Triton X-100, followed by incubation with fluorophore-conjugated secondary antibodies in 0.3% Triton X-100 and 10% donkey serum for 2 h at room temperature. Sections were rinsed and mounted onto slides for imaging. The following antibodies were used: rabbit anti-c-Fos (1:3000, Ab190289, Abcam, Cambridge, UK), rabbit anti–NeuN (1:1000, 26975-1-AP, Proteintech, Rosemont, IL, USA), donkey anti-rabbit Cy3 (1:1000, 711-165-152, Jackson ImmunoResearch, West Grove, PA, USA), and donkey anti-rabbit Alexa Fluor 488 (1:1000, 711-545-152, Jackson ImmunoResearch).

### 2.8. Cell Counting

Fluorescence images of immunostained brain sections were acquired using a VS200 slide scanner (Olympus, Tokyo, Japan). Coronal sections were registered to the standard mouse brain atlas (Paxinos and Franklin, 2013 [27]) using anatomical landmarks such as the third ventricle (3V) for alignment. c-Fos-positive neurons located within the defined regions of interest (ROIs) were manually quantified using ImageJ software (version 1.54f, Fiji distribution; National Institutes of Health, Bethesda, MD, USA).

### 2.9. Statistical Analysis

All statistical analyses were performed using GraphPad Prism (version 9.5, GraphPad Software, San Diego, CA, USA). Figures were prepared using Adobe Photoshop and Adobe Illustrator (version 2021, Adobe Inc., San Jose, CA, USA). Quantitative data are presented as the mean ± standard error of the mean (SEM). Statistical tests were selected based on data distribution and experimental design to ensure appropriate and rigorous analysis. Comparisons between two independent groups were performed using unpaired, two-tailed Student’s *t*-tests. For multiple-group comparisons, one-way or two-way ANOVA followed by post hoc tests were applied. A *p*-value < 0.05 was considered statistically significant for all tests. All analyses were performed by investigators blinded to experimental group assignments.

## 3. Results

### 3.1. Chronic Sleep Disruption Alters Sleep–Wake States and Enhances Sleep Rebound

**Figure 1:**
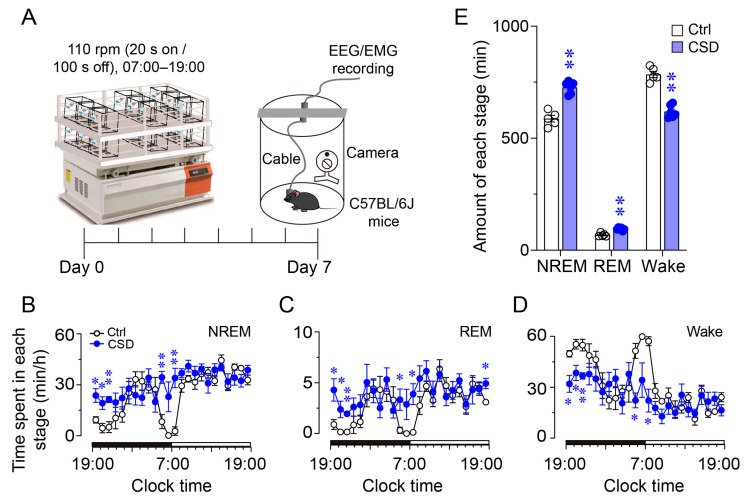
Chronic sleep disruption alters sleep–wake states in mice. (**A**) Schematic representation of the chronic sleep disruption (CSD) protocol. Mice were subjected to a 7-day CSD protocol using an automated orbital shaker operating at 110 rpm in cycles of 20 s on and 100 s off, from 07:00 to 19:00. EEG/EMG recordings were performed in C57BL/6J mice housed in video-monitored recording chambers for a 24 h period after 7 consecutive days of sleep disturbance, aiming to evaluate changes in sleep architecture. (**B**–**D**) Hourly distribution of time spent in NREM sleep (**B**), REM sleep (**C**), and wakefulness (**D**) across the 24 h cycle in control (Ctrl, *n* = 5) and CSD (*n* = 6) groups. Statistical analysis was performed using repeated-measures ANOVA with multiple comparisons corrected by the false discovery rate (FDR): NREM, *F*(1,9) = 59.67, *p* < 0.001; REM, *F*(1,9) = 41.23, *p* = 0.001; Wake, *F*(1,9) = 84.36, *p* < 0.0001. Data are presented as mean ± SEM. * *p* < 0.05, ** *p* < 0.01. (**E**) Total time spent in NREM sleep, REM sleep, and wakefulness during a 24 h recording period in control and CSD groups (Ctrl, *n* = 5; CSD, *n* = 6). Group differences were assessed using unpaired two-tailed Student’s *t*-tests: NREM sleep, *t*(9) = 7.72, *p* = 2.9 × 10^−5^; REM sleep, *t*(9) = 6.42, *p* = 1.22 × 10^−4^; Wake, *t*(9) = 9.19, *p* = 7.0 × 10^−6^. Data are presented as mean ± SEM. ** *p* < 0.01.

To investigate the effects of chronic sleep disruption (CSD) on c-Fos expression within the deep cerebellar nuclei (DCN), we employed a 7-day sleep disruption paradigm using an orbital shaker in adult male C57BL/6J mice (8–12 weeks old). EEG and EMG electrodes were implanted, and animals were allowed to recover for at least one week before being randomly assigned to either the CSD group or control group. The CSD group underwent repeated disturbance during the light phase (Figure 1A), while controls were housed on standard racks in the same room without additional intervention. Continuous EEG/EMG recordings were performed for 24 h immediately after termination of the CSD protocol to evaluate sleep–wake state alterations and subsequent rebound sleep.

Mice subjected to CSD exhibited a significant increase in both NREM and REM sleep during the dark phase, particularly within the first 3 h following the termination of sleep disturbance. In contrast, during the light phase, only the initial time point after lights-on showed a transient elevation in NREM sleep, with no significant changes observed thereafter (Figure 1B–D). Quantitative analysis of cumulative 24-h sleep–wake amounts revealed that NREM sleep increased from 587.6 ± 14.2 min to 726.1 ± 10.6 min, REM sleep increased from 68.3 ± 3.0 min to 94.9 ± 2.4 min, and wakefulness decreased from 784.9 ± 13.4 min to 618.7 ± 10.3 min (Figure 1E). These results demonstrate that chronic sleep disturbance significantly altered sleep architecture in mice, supporting the successful establishment of the CSD model.

### 3.2. Chronic Sleep Disruption Impairs Locomotor Activity and Motor Coordination

**Figure 2:**
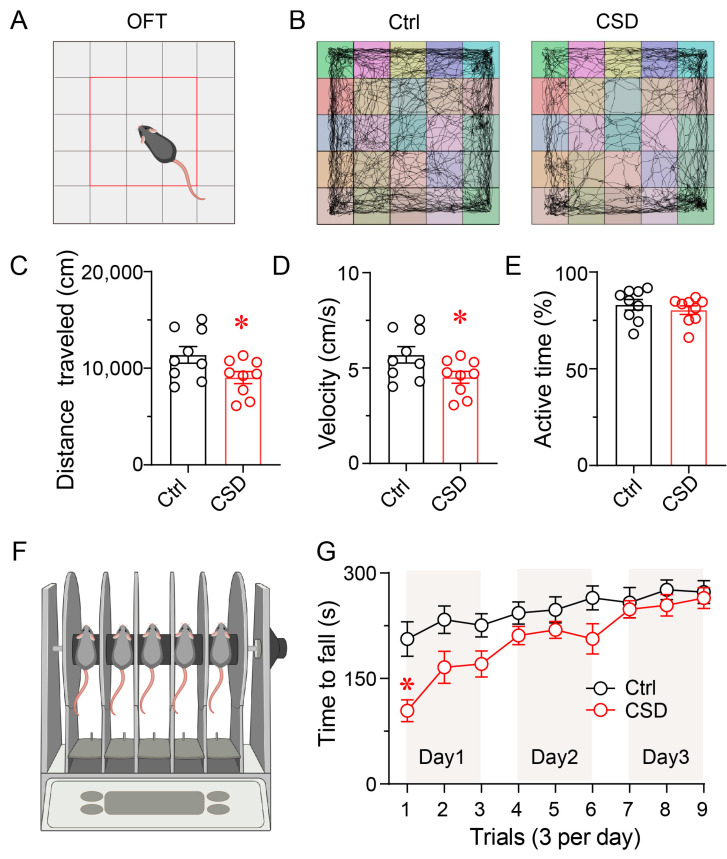
Chronic sleep disruption impairs locomotor activity and motor coordination. (**A**) Schematic diagram of the open field test (OFT) used to assess locomotor behavior. (**B**) Representative movement trajectories of control mice (left) and CSD mice (right) during the 30 min OFT. (**C**–**E**) Quantification of locomotor parameters during the 30 min OFT. Compared with controls, CSD mice showed a significant reduction in total distance traveled (**C**), average velocity (**D**), whereas no significant difference was observed in percentage of active time (**E**). Data are presented as mean ± SEM (*n* = 9 per group). Unpaired two-tailed *t*-tests: C: *t*(16) = 2.229, *p* = 0.0405; D: *t*(16) = 2.228, *p* = 0.041; E: *t*(16) = 0.797, *p* = 0.437. * *p* < 0.05. (**F**) Schematic representation of the rotarod apparatus used to assess motor coordination and learning. (**G**) Rotarod performance across three consecutive testing days (three trials per day). On Day 1, CSD mice exhibited a significantly shorter latency to fall compared to controls. Performance gradually improved across days in both groups, and no significant differences were detected on Days 2 and 3. Data are presented as mean ± SEM (*n* = 9 per group). Two-way repeated-measures ANOVA followed by Sidak’s post hoc test, *F*(1,16) = 6.516, *p* = 0.0213. * *p* < 0.05.

To evaluate the behavioral consequences of CSD, open-field and rotarod tests were conducted one day after completion of the 7-day CSD paradigm to assess spontaneous locomotion and motor coordination. In the open-field test, representative movement trajectories revealed reduced exploratory behavior in CSD mice compared to controls (Figure 2A,B). Quantitative analysis showed that CSD mice exhibited a 21% reduction in both total distance traveled and average velocity (*p* < 0.05), whereas the percentage of active time was not significantly altered compared with controls (Figure 2C–E).

Motor coordination and learning ability were further evaluated using the accelerating rotarod test across three consecutive days (three trials per day). On Day 1, CSD mice showed a significantly shorter latency to fall relative to controls (*p* < 0.05), indicating impaired motor coordination. Performance improved across subsequent days in both groups, and no significant differences were detected on Days 2 and 3 (Figure 2F,G). These findings suggest that chronic sleep disruption reduces locomotor activity and transiently impairs motor coordination, while motor learning ability remains intact.

### 3.3. Chronic Sleep Disruption Selectively Activates Neurons in the Lateral Nucleus

**Figure 3:**
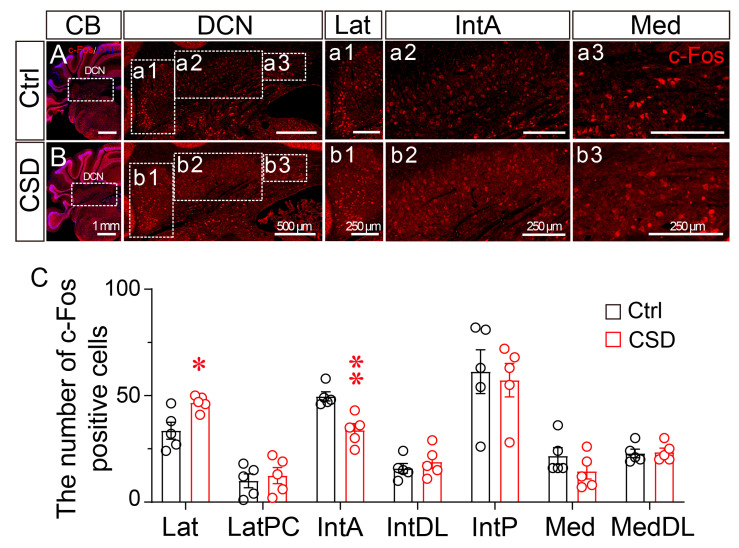
Chronic sleep disruption alters the pattern of c-Fos expression in the deep cerebellar nuclei. (**A**,**B**) Representative immunofluorescence images showing c-Fos expression (red) in the cerebellum (CB) and deep cerebellar nuclei (DCN) of control (Ctrl, (**A**)) and chronic sleep disruption (CSD, (**B**)) mice. Dotted boxes indicate the regions of interest corresponding to the lateral (Lat), interposed anterior (IntA), and medial (Med) nuclei, which are shown at higher magnification in (**a1**–**a3**) and (**b1**–**b3**). DAPI (blue) was used for nuclear counterstaining. Scale bars: 1 mm in CB overview images; 500 μm in DCN overview images; 250 μm in high-magnification panels. (**C**) Quantification of c-Fos-positive cell numbers across DCN subregions, including the lateral (Lat), lateral parvicellular (LatPC), interposed anterior (IntA), interposed dorsolateral (IntDL), interposed posterior (IntP), medial (Med), and medial dorsolateral (MedDL) nuclei. Data are presented as mean ± SEM (*n* = 5 per group). Statistical comparisons were performed using unpaired two-tailed *t*-tests: Lat, *t*(8) = 3.06, *p* = 0.015; LatPC, *t*(8) = 0.48, *p* = 0.64; IntA, *t*(8) = 4.21, *p* = 0.003; IntDL, *t*(8) = 0.77, *p* = 0.459; IntP, *t*(8) = 0.31, *p* = 0.765; Med, *t*(8) = 1.36, *p* = 0.212; MedDL, *t*(8) = 0.22, *p* = 0.832. * *p* < 0.05, ** *p* < 0.01.

To determine whether CSD alters neuronal activity within the deep cerebellar nuclei, immunofluorescence staining for c-Fos, an immediate early gene commonly used as a marker of neuronal activation, was performed following the 7-day sleep disruption protocol. c-Fos expression was quantified across distinct DCN subregions. Representative images revealed that CSD markedly increased c-Fos expression in the lateral nucleus (Lat) compared to controls (Figure 3A,B(a1–3,b1–3)).

Quantitative analysis revealed a 1.39-fold increase in c-Fos-positive neurons in the lateral nucleus (*p* < 0.05), whereas the interposed anterior nucleus (IntA) exhibited a 32% reduction relative to controls (*p* < 0.01). In contrast, no significant changes were detected in the posterior interposed (IntP), dorsolateral interposed (IntDL), medial (Med), dorsolateral medial (MedDL), or lateral parvicellular (LatPC) nuclei (Figure 3C). These findings indicate that chronic sleep disruption preferentially activates neuronal populations in the lateral nucleus, suggesting their potential involvement in sleep–wake-related motor regulation.

### 3.4. Chemogenetic Activation of Lat^Vglut2+^ Neurons Reduces Locomotor Activity and Motor Coordination

**Figure 4:**
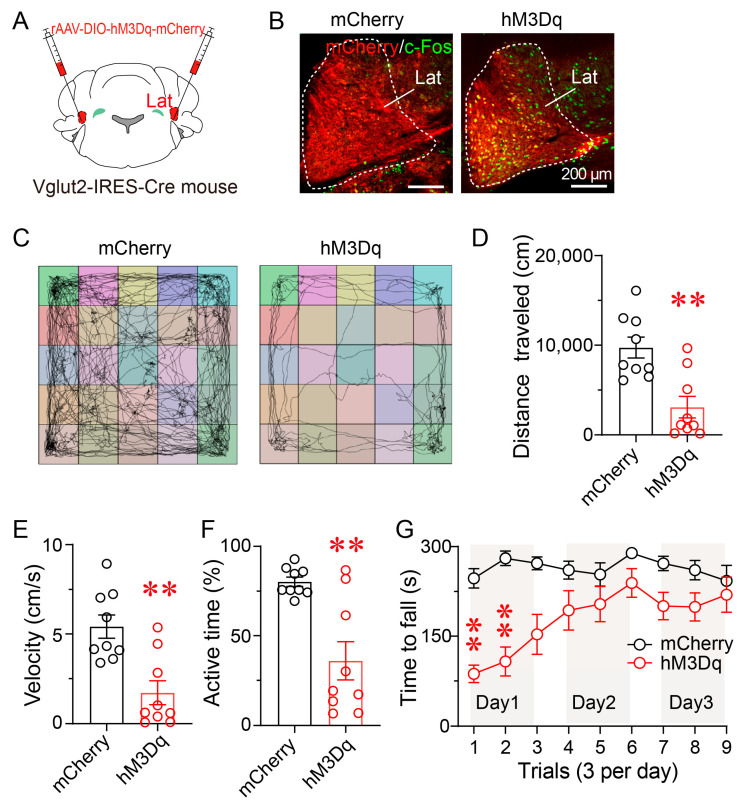
Chemogenetic activation of Lat^Vglut2+^ neurons reduces locomotor activity and motor coordination. (**A**) Schematic of bilateral stereotaxic injection of rAAV-DIO-hM3Dq-mCherry into the Lat of Vglut2-IRES-Cre mice. (**B**) Representative immunofluorescence images showing expression of hM3D(Gq)-mCherry (red) and c-Fos (green) in the Lat of Vglut2-IRES-Cre mice. Compared with mCherry controls (left), hM3Dq-expressing mice (right) exhibited robust c-Fos expression following CNO administration, confirming chemogenetic activation of Lat^Vglut2+^ neurons. White dashed lines outline the boundaries of the lateral nucleus. Scale bar: 200 μm. (**C**) Representative movement trajectories from mCherry or hM3Dq mice during the 30 min OFT. (**D**–**F**) Quantification of locomotor parameters during the 30 min OFT. Compared with controls, hM3Dq mice showed a significant reduction in total distance traveled (**D**), average velocity (**E**), and active time (**F**). Data are presented as mean ± SEM (*n* = 9 per group). Unpaired two-tailed *t*-tests: (**D**) *t*(16) = 3.960, *p* = 0.0011; (**E**) *t*(16) = 3.960, *p* = 0.0011; (**F**) *t*(16) = 4.029, *p* = 0.0010. ** *p* < 0.01. (**G**) Rotarod performance over three consecutive days (three trials per day). On Day 1, hM3Dq-expressing mice exhibited a significantly shorter latency to fall compared to controls. Performance progressively improved across subsequent days in both groups. Data are presented as mean ± SEM (*n* = 9 per group). Two-way repeated-measures ANOVA with Sidak’s post hoc test, *F*(1,16) = 14.72, *p* = 0.0015. ** *p* < 0.01.

The lateral nucleus is primarily composed of glutamatergic neurons, which serve as the principal excitatory output from the DCN to key motor-related regions—including the motor cortex, red nucleus, and thalamus—and play a central role in motor planning, coordination, and voluntary movement control. To test whether selective activation of glutamatergic neurons in lateral nucleus (Lat^Vglut2+^) is sufficient to reproduce the motor impairments induced by CSD, we employed a chemogenetic activation strategy. Vglut2-IRES-Cre mice received bilateral injections of rAAV-DIO-hM3Dq-mCherry into the Lat, enabling Cre-dependent expression of the excitatory DREADD receptor hM3Dq (Figure 4A). Immunofluorescence analysis confirmed robust expression of hM3Dq-mCherry and c-Fos in the targeted region following CNO administration, indicating successful activation of glutamatergic neurons (Figure 4B).

In the open field test, hM3Dq-expressing mice exhibited a significant reduction in locomotor activity, with total distance traveled and average velocity both reduced by 68%, and active time decreased by 55% compared to mCherry controls (*p* < 0.01; Figure 4C–F). These results demonstrate that activation of Lat^Vglut2+^ neurons reduces spontaneous locomotor activity.

Motor coordination was further evaluated using the accelerating rotarod test. On Day 1, hM3Dq-expressing mice showed a significantly shorter latency to fall relative to controls (*p* < 0.01), reflecting impaired motor coordination. Performance gradually improved over the following two days, and no significant differences were observed between groups on Day 2 or Day 3 (Figure 4G). These results indicate that chemogenetic activation of Lat^Vglut2+^ neurons impairs motor coordination without affecting motor learning.

### 3.5. Ablation of Lat^Vglut2+^ Neurons Impairs Locomotor Activity and Motor Coordination

**Figure 5:**
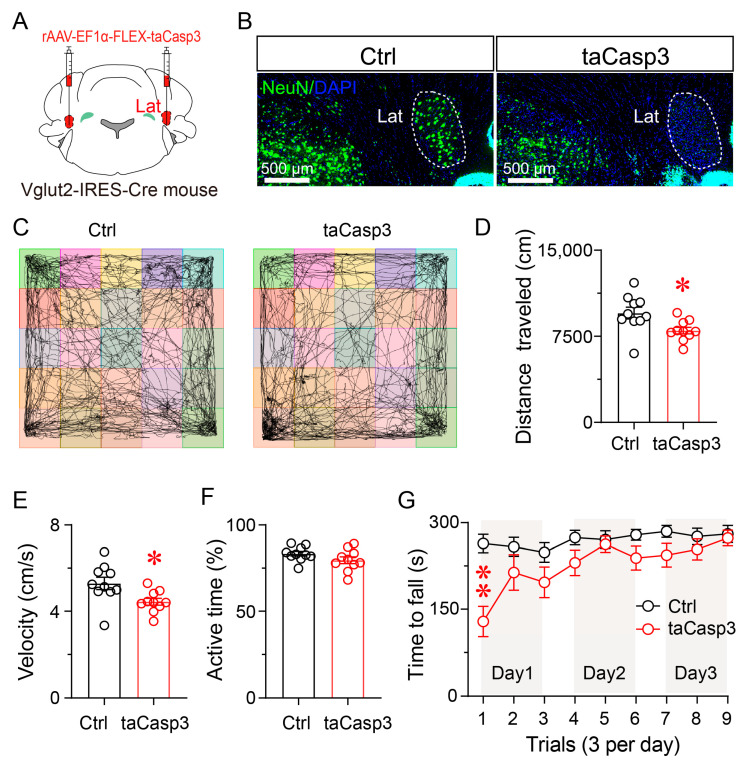
Selective ablation of Lat^Vglut2+^ neurons impairs locomotor performance and motor coordination. (**A**) Schematic of bilateral stereotaxic injection of rAAV-EF1α-FLEX-taCasp3 into the Lat of Vglut2-IRES-Cre mice to induce Cre-dependent neuronal ablation. (**B**) Representative immunofluorescence images showing NeuN (green) and DAPI (blue) staining in the Lat of control and taCasp3-treated mice, confirming neuronal loss following taCasp3 expression. White dashed lines outline the boundaries of the lateral nucleus. Scale bars: 500 μm. (**C**) Representative movement trajectories of control and taCasp3-treated mice in the 30 min OFT. (**D**–**F**) Quantification of locomotor activity during the 30 min OFT. Compared to controls, taCasp3-treated mice exhibited significantly reduced total distance traveled (**D**) and average velocity (**E**), whereas the percentage of active time (**F**) was not significantly different. Data are presented as mean ± SEM (*n* = 10 per group). Unpaired two-tailed *t*-tests: (**D**) *t*(18) = 2.580, *p* = 0.019; (**E**) *t*(18) = 2.592, *p* = 0.018; (**F**) *t*(18) = 1.584, *p* = 0.131. * *p* < 0.05. (**G**) Rotarod performance across three consecutive days (three trials per day). On Day 1, taCasp3-treated mice exhibited significantly shorter latency to fall compared to controls. Motor performance gradually improved over subsequent trials in both groups. Data are presented as mean ± SEM (*n* = 11 per group). Two-way repeated-measures ANOVA with Sidak’s post hoc test, *F*(1,20) = 10.07, *p* = 0.0048. ** *p* < 0.01.

To evaluate the necessity of Lat^Vglut2+^ neurons in motor regulation, we selectively ablated these neurons using a Cre-dependent caspase-3 strategy. Vglut2-IRES-Cre mice received bilateral injections of rAAV-EF1α-FLEX-taCasp3 into the lateral nucleus, resulting in targeted apoptosis of glutamatergic neurons (Figure 5A). Immunofluorescence staining for NeuN, a marker of mature neurons, confirmed a marked reduction in neuronal density in taCasp3-injected mice compared to controls (Figure 5B), verifying the efficacy of the ablation strategy.

In the open-field test, taCasp3-treated mice exhibited a 16.59% reduction in total distance traveled and a 16.63% reduction in average velocity (both *p* < 0.05) (Figure 5C–E), while the percentage of active time was not significantly altered compared to controls (Figure 5F). These findings suggest that ablation of Lat^Vglut2+^ neurons leads to reduced spontaneous locomotion.

In the rotarod test, taCasp3-treated mice showed a significantly shorter latency to fall compared to controls on Day 1 (*p* < 0.05), indicating impaired motor coordination. Performance improved over subsequent trials, and no significant differences were observed between groups on Day 2 or Day 3 (Figure 5G). This pattern suggests that while the loss of glutamatergic neurons in the lateral nucleus compromises initial motor coordination, the capacity for motor learning remains largely intact.

### 3.6. Ablation of Lat^Vglut2+^ Neurons Does Not Induce Anxiety-like Behavior or Impair Spatial Working Memory

To investigate whether Lat^Vglut2+^ neurons contribute to emotional or cognitive functions beyond motor control, we assessed anxiety-like behavior and spatial working memory following taCasp3-mediated ablation using the elevated plus maze (EPM) and Y-maze paradigms.

**Figure 6:**
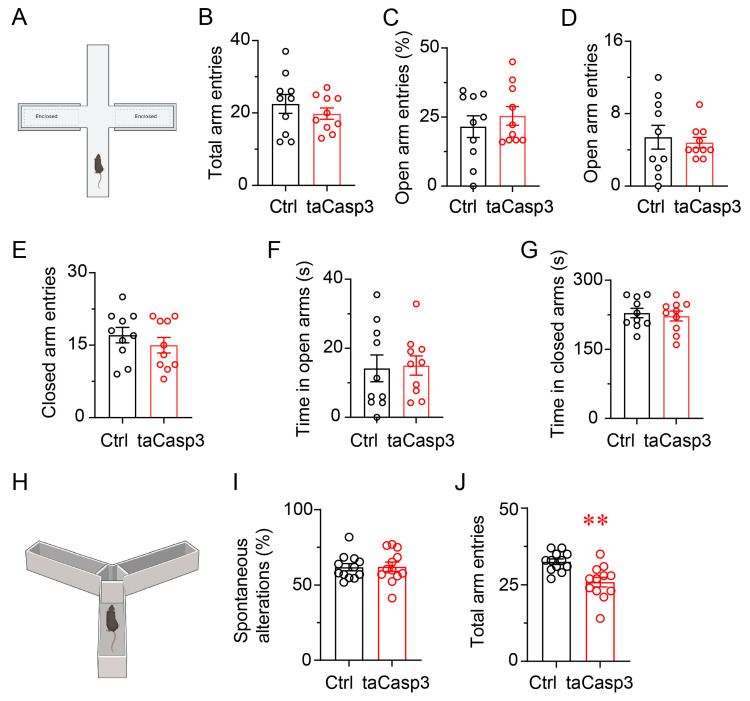
Selective ablation of Lat^Vglut2+^ neurons does not affect anxiety-like behavior or spatial working memory. (**A**) Schematic of the elevated plus maze (EPM) used to assess anxiety-like behavior. (**B**–**G**) Quantification of EPM parameters, including total arm entries (**B**), percentage of open arm entries (**C**), number of open arm entries (**D**), number of closed arm entries (**E**), time spent in open arms (**F**), and time spent in closed arms (**G**). No significant differences were observed between control and taCasp3-treated mice, indicating that ablation of Lat^Vglut2+^ neurons did not alter anxiety-like behavior. Data are presented as mean ± SEM (*n* = 10 per group). Unpaired two-tailed *t*-tests: (**B**) *t*(18) = 0.886, *p* = 0.387; (**C**) *t*(18) = 0.754, *p* = 0.461; (**D**) *t*(18) = 0.417, *p* = 0.681; (**E**) *t*(18) = 0.9284, *p* = 0.366; (**F**) *t*(18) = 0.170, *p* = 0.867; (**G**) *t*(18) = 0.440, *p* = 0.665. (**H**) Schematic of the Y-maze used to evaluate spatial working memory. (**I**,**J**) Quantification of spontaneous alternation percentage (**I**) and total arm entries (**J**) during the Y-maze test. taCasp3-treated mice exhibited significantly fewer total arm entries, while spontaneous alternation was unaffected. Data are presented as mean ± SEM (*n* = 12 per group). Unpaired two-tailed *t*-tests: (**I**): *t*(22) = 0.089, *p* = 0.930; (**J**): *t*(22) = 3.694, *p* = 0.001. ** *p* < 0.01.

In the EPM (Figure 6A), taCasp3-treated mice did not differ from controls in total arm entries, percentage of open arm entries, number of open and closed arm entries, or time spent in open and closed arms (all *p* > 0.05; Figure 6B–G). These results suggest that ablation of Lat^Vglut2+^ neurons does not induce anxiety-like behavior.

In the Y-maze test (Figure 6H), the percentage of spontaneous alternations, a widely used index of spatial working memory, was not significantly affected by taCasp3 treatment (*p* > 0.05, Figure 6I). However, taCasp3-treated mice showed a significant reduction in total arm entries compared with controls (*p* < 0.01, Figure 6I), indicating reduced general exploratory drive rather than a deficit in working memory. These results demonstrate that Lat^Vglut2+^ neurons are essential for motor performance, but they appear to play a minimal role in regulating anxiety-related behavior or spatial working memory under baseline conditions.

### 3.7. Chemogenetic Inhibition of Lat^Vglut2+^ Neurons Attenuated CSD-Induced Impairments in Locomotor Activity and Motor Coordination

**Figure 7:**
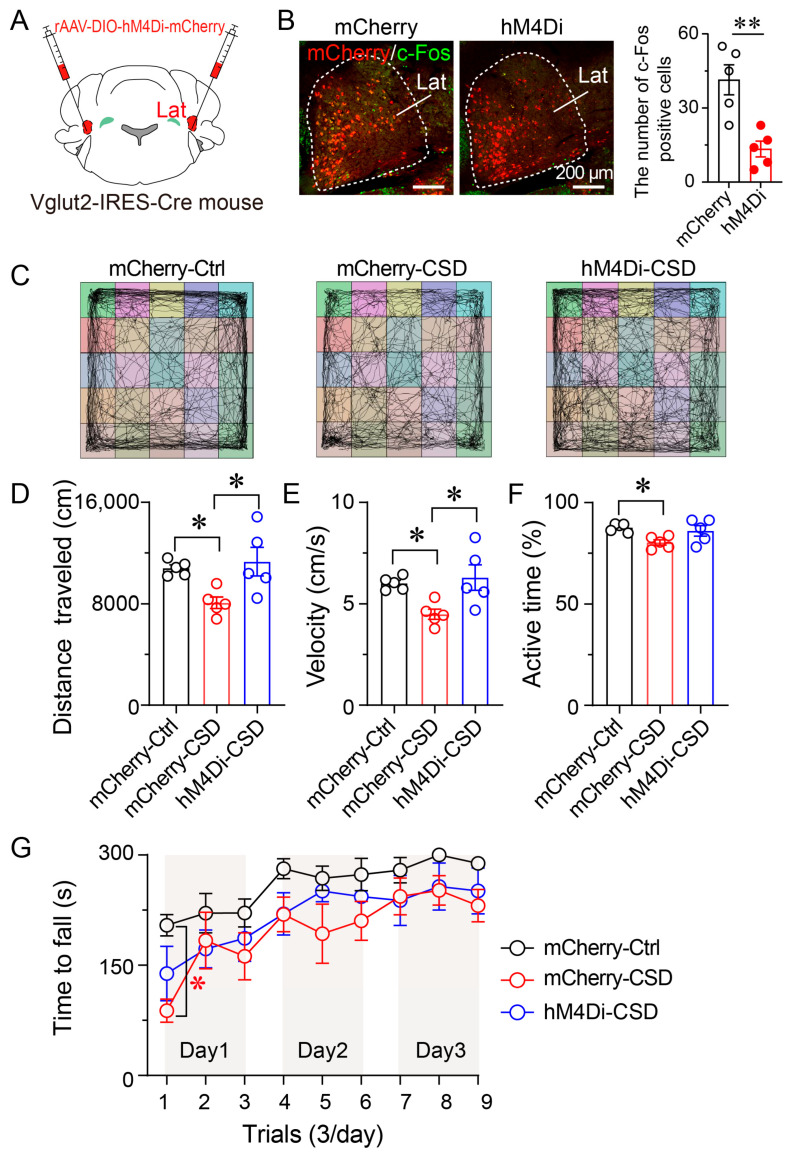
Chemogenetic inhibition of Lat^Vglut2+^ neurons attenuates CSD-induced motor deficits. (**A**) Schematic representation of bilateral stereotaxic injection of rAAV-DIO-hM4Di-mCherry into the Lat of Vglut2-IRES-Cre mice. (**B**) Representative immunofluorescence images showing mCherry (red) and c-Fos (green) expression in control (mCherry) and hM4Di-expressing mice. White dashed lines delineate the boundaries of the Lat. Quantification of c-Fos-positive cells revealed a significant reduction in neuronal activity in the hM4Di group (red circles) compared with the mCherry control group (white circles). Data are presented as mean ± SEM (*n* = 5 per group). Unpaired two-tailed *t*-tests: t (4) = 4.079, *p* = 0.004. ** *p* < 0.01. Scale bar: 200 μm. (**C**) Representative movement trajectories of mice from mCherry-Ctrl, mCherry-CSD, and hM4Di-CSD groups during the 30 min OFT. (**D**–**F**) Quantification of locomotor activity during the 30 min OFT. Compared with mCherry-CSD mice, hM4Di-expressing CSD mice exhibited significantly increased total distance traveled (**D**), average velocity (**E**), while the percentage of active time showed a non-significant increasing trend (**F**). Data are presented as mean ± SEM (*n* = 5 per group), One-way ANOVA with Tukey’s post hoc test. * *p* < 0.05. (**G**) Rotarod performance across three consecutive days (three trials per day). On Day 1, hM4Di-expressing CSD mice displayed longer latency to fall compared with mCherry-CSD control. No significant group differences were detected on Days 2–3. Data are presented as mean ± SEM (*n* = 5 per group). Two-way repeated-measures ANOVA with Tukey’s post hoc test, * *p* < 0.05.

To determine whether chemogenetic inhibition of Lat^Vglut2+^ neurons could alleviate motor impairments induced by CSD, Vglut2-IRES-Cre mice received bilateral injections of either rAAV-DIO-hM4Di-mCherry or control rAAV-DIO-mCherry into the lateral nucleus (Figure 7A). After allowing three weeks for viral expression, mice were divided into three groups: (1) mCherry-Ctrl (control, no CSD), (2) mCherry-CSD (control virus + CSD), and (3) hM4Di-CSD (hM4Di + CSD). Both CSD groups (mCherry-CSD and hM4Di-CSD) were subjected to a 7-day CSD paradigm using an orbital platform shaker, during which mice received daily intraperitoneal injections of CNO (1 mg/kg) at 9:00 a.m. Behavioral testing was conducted after the completion of 7-day CSD. Immunofluorescence analysis confirmed robust hM4Di-mCherry expression within the lateral nucleus. Following CNO administration, c-Fos immunoreactivity was markedly reduced in hM4Di-expressing mice compared with mCherry controls (*p* < 0.01, Figure 7B), indicating that chemogenetic manipulation effectively suppressed the activity of glutamatergic neurons in the lateral nucleus.

In the open field test, CSD mice expressing hM4Di exhibited significantly higher locomotor activity compared with mCherry-CSD controls, as reflected by increased total distance traveled (*p* < 0.01), and average velocity (*p* < 0.01), while the percentage of active time exhibited a non-significant increasing trend (Figure 7C–F).

In the rotarod test, on Day 1, mCherry-CSD mice exhibited a significantly shorter latency to fall compared with mCherry-Ctrl, confirming impaired motor coordination. hM4Di-CSD mice showed intermediate performance between mCherry-CSD and mCherry-Ctrl, although the difference relative to mCherry-CSD did not reach statistical significance. Over subsequent trials (Days 2–3), all groups improved with training, and no significant differences were detected among groups (Figure 7G). Taken together with the OFT results, these findings suggest that inhibition of Lat^Vglut2+^ neurons partially alleviates CSD-induced locomotor and coordination deficits.

## 4. Discussion

In this study, we demonstrated that CSD impairs motor behavior, particularly locomotor activity and motor coordination. A 7-day CSD paradigm selectively increased c-Fos expression in the lateral nucleus of the DCN. Moreover, chemogenetic activation or ablation of Lat^Vglut2+^ neurons disrupted motor coordination and reduced locomotor activity, whereas chemogenetic inhibition of these neurons attenuated CSD-induced motor deficits. Together, these findings identify Lat^Vglut2+^ neurons as a critical cerebellar population mediating sleep-related motor dysfunction.

Sleep is well known to facilitate motor performance [28,29,30], and both our previous work [8] and the present study demonstrated that a 7-day CSD protocol diminishes exploratory behavior and impairs motor coordination. Accumulating evidence indicates that the cerebellum contributes to both motor control and sleep–wake regulation [10,31]; however, the specific subregions and cellular substrates underlying this interaction remain poorly defined. Neuroimaging and clinical studies have revealed cerebellar abnormalities in patients with sleep disorders such as insomnia and sleep apnea, implicating cerebellum as a potential hub linking sleep and motor function [31,32]. Nevertheless, most of these findings have been correlative. Here, we identify a discrete population of excitatory Lat^Vglut2+^ neurons that mediates motor impairment following CSD. Our results provide the first direct causal evidence that aberrant activation of Lat^Vglut2+^ neurons is sufficient to induce motor deficits resembling those observed by CSD, thereby positioning the lateral nucleus as a key mechanistic link in sleep–motor integration.

The lateral nucleus is the largest and phylogenetically most recent of the deep cerebellar nuclei [14]. It plays a critical role in planning, initiating, and fine-tuning voluntary movements through excitatory outputs to motor-related thalamic and cortical regions [14]. In our study, CSD selectively increased neuronal activity in the lateral nucleus, while sparing or even reducing activity in other DCN subregions, suggesting a region-specific cerebellar response to CSD. Chemogenetic activation of Lat^Vglut2+^ neurons induced immediate deficits in motor coordination, mimicking the behavioral consequences of CSD. Similarly, selective ablation of Lat^Vglut2+^ neurons resulted in reduced spontaneous locomotion and impaired rotarod performance, indicating that both excessive and diminished output from this population disrupt motor function. Notably, ablation of Lat^Vglut2+^ neurons had minimal impact on anxiety-like behavior or working memory, suggesting a motor-specific functional role of this population. Together, these findings suggest that sleep disruption may preferentially perturb cerebellar circuits involved in motor performance, and highlight the lateral nucleus as a selectively modulated structure in response to chronic sleep disruption.

As the primary output structure of the cerebellum, the DCN receive Purkinje cell inhibitory input [33,34]. Sleep dynamically modulates cerebellar activity, including state-dependent changes in Purkinje cell firing patterns and afferent inputs [35,36]. CSD may disrupt this balance, leading to diminished inhibitory drive and disinhibited excitatory output of downstream Lat^Vglut2+^ neurons. In the context of CSD, we propose that, under CSD conditions, sleep loss may reduce Purkinje-mediated inhibition—possibly through altered oscillatory coupling or neuromodulatory changes—thereby enhancing the excitability of Lat^Vglut2+^ neurons. This hypothesis is supported by our chemogenetic activation experiments, in which enhanced lateral nucleus output recapitulated CSD-induced motor impairments and hypoactivity. Comparable disinhibitory mechanisms have been described in cerebellar ataxia models, where compromised Purkinje output leads to DCN hyperexcitability and motor dysfunction [34,37,38]. Our findings that both activation and ablation of LatVglut2+ neurons impaired motor coordination indicate that lateral nucleus function depends critically on the precise regulation of intrinsic excitatory output. This regulation, mediated by Purkinje cell inhibition and the intrinsic excitability of glutamatergic neurons, is essential for maintaining cerebellar motor control. Disruption of this delicate regulatory mechanism may lead to significant motor coordination deficits. This interpretation is consistent with classical models of cerebellar function, in which Purkinje cells regulate DCN excitability to shape motor output, and disruptions in this inhibitory control result in abnormal movement. Future studies will assess synaptic inhibition or Purkinje–DCN interactions using simultaneous recordings of Purkinje cell activity and Lat^Vglut2+^ neuron firing under CSD conditions.

Despite the clarity of the results, several limitations of our study should be acknowledged. Although the orbital shaker paradigm was optimized to minimize mechanical disturbance, we cannot completely exclude minor equilibrium-related influences. Future studies employing alternative paradigms, such as gentle-handling or dark-phase interventions, will be necessary to further rule out potential vestibular or sleep-unrelated effects. Although c-Fos immunolabeling revealed robust neuronal activation within the lateral nucleus following CSD, the specific cellular identities of these activated neurons were not directly verified. Nevertheless, the consistent behavioral effects produced by chemogenetic manipulation of Lat^Vglut2+^ neurons suggest that the majority of c-Fos-positive cells are glutamatergic. It is also important to note that c-Fos immunolabeling captures only a static representation of neuronal activity. Elucidating how sleep disruption dynamically alters firing patterns across brain states will require in vivo electrophysiology or calcium imaging in lateral nucleus neurons and their Purkinje inputs. Although our analyses primarily focus on the lateral nucleus due to its pronounced c-Fos elevation, we also observed a reduction in c-Fos expression in the anterior interposed nucleus. The functional significance of this decrease remains unclear, but it may reflect compensatory suppression in a parallel motor circuit or altered cerebellar network interactions. Further studies should investigate how other DCN subregions and cerebellar cortical areas contribute to sleep disruption-induced behavioral phenotypes. In particular, identifying the cortical zones and Purkinje subpopulations drive lateral nucleus hyperactivity during CSD could help establish a more comprehensive mechanistic framework of sleep-related cerebellar dysfunction.

From an evolutionary perspective, the cerebellar lateral nucleus corresponds to the human dentate nucleus, which represents the most recently evolved subdivision of the DCN Comparative transcriptomic analyses have shown that cerebellar nuclei evolved through repeated duplication of a conserved cell-type set [39]. Although the human dentate nucleus is markedly expanded and exhibits preferential connectivity with prefrontal and associative cortices, its core glutamatergic output architecture is conserved across mammals. This evolutionary conservation suggests that the fundamental mechanism by which lateral/dentate glutamatergic neurons regulate motor co-ordination is likely shared across species. However, in humans, the expansion and enhanced prefrontal coupling of the dentate nucleus may further contribute to cognitive consequences of sleep disruption, highlighting both the evolutionary continuity and translational significance of cerebellar mechanisms in sleep-related motor and cognitive regulation.

In conclusion, we identify the lateral nucleus that links sleep disruption to motor dysfunction, thereby providing new insight into how physiological states perturb motor circuits. These findings refine our understanding of the cerebellum’s contribution to motor impairments associated with sleep-related disorders, including insomnia, sleep apnea, and circadian rhythm disturbances. The lateral nucleus emerges as a key node in sleep-dependent modulation of motor control. These results expand the functional repertoire of the cerebellum, highlighting its integrative role in encoding internal brain states and relaying them to motor effectors [40]. From a translational perspective, Lat^Vglut2+^ may represent a potential therapeutic target. Future studies should explore neuromodulatory approaches, such as cerebellar transcranial stimulation or pharmacological modulation of cerebellar excitability, to mitigate motor symptoms associated with chronic sleep loss.

## 5. Conclusions

This study demonstrates that chronic sleep disruption selectively increased neuronal activity in the cerebellar lateral nucleus and impaired locomotor activity and motor coordination. Chemogenetic activation or ablation of Lat^Vglut2+^ neurons reproduced motor deficits, while inhibition attenuated CSD-induced impairments, highlighting the bidirectional sensitivity of motor function to lateral nucleus excitatory output. These findings provide direct causal evidence that hyperactivity of Lat^Vglut2+^ neurons mediates motor dysfunction following chronic sleep disruption. Importantly, this work identifies the cerebellar lateral nucleus as a mechanistic link between sleep and motor control, suggesting novel therapeutic targets for sleep-related motor disorders.

## Data Availability

The data and analyses used in this study can be made available from the corresponding author upon reasonable request. The data are not publicly available due to the large volume of raw files (e.g., EEG/EMG recordings and behavioral videos) and institutional data storage limitations.

## Abbreviations

The following abbreviations are used in this manuscript:

ANOVA	analysis of variance
CSD	chronic sleep disruption
DCN	deep cerebellar nuclei
EEG	electroencephalogram
EMG	electromyogram
IntA	interposed anterior nucleus
IntDL	dorsolateral interposed nucleus
IntP	posterior interposed nucleus
Lat	lateral nucleus
Med	medial nucleus
MedDL	dorsolateral medial nucleus
NREM	non-rapid eye movement
REM	rapid eye movement
